# Targeting of the Human Coagulation Factor IX Gene at rDNA Locus of Human Embryonic Stem Cells

**DOI:** 10.1371/journal.pone.0037071

**Published:** 2012-05-16

**Authors:** Xionghao Liu, Yong Wu, Zhuo Li, Junlin Yang, Jinfeng Xue, Youjin Hu, Mai Feng, Wenbin Niu, Qiurui Yang, Ming Lei, Jiahui Xia, Lingqian Wu, Desheng Liang

**Affiliations:** State Key Laboratory of Medical Genetics, Central South University, Changsha, Hunan, China; Instituto de Medicina Molecular, Portugal

## Abstract

**Background:**

Genetic modification is a prerequisite to realizing the full potential of human embryonic stem cells (hESCs) in human genetic research and regenerative medicine. Unfortunately, the random integration methods that have been the primary techniques used keep creating problems, and the primary alternative method, gene targeting, has been effective in manipulating mouse embryonic stem cells (mESCs) but poorly in hESCs.

**Methodology/Principal Findings:**

Human ribosomal DNA (rDNA) repeats are clustered on the short arm of acrocentric chromosomes. They consist of approximately 400 copies of the 45S pre-RNA (rRNA) gene per haploid. In the present study, we targeted a physiological gene, human coagulation factor IX, into the rDNA locus of hESCs via homologous recombination. The relative gene targeting efficiency (>50%) and homologous recombination frequency (>10^−5^) were more than 10-fold higher than those of loci targeted in previous reports. Meanwhile, the targeted clones retained both a normal karyotype and the main characteristics of ES cells. The transgene was found to be stably and ectopically expressed in targeted hESCs.

**Conclusion/Significance:**

This is the first targeting of a human physiological gene at a defined locus on the hESC genome. Our findings indicate that the rDNA locus may serve as an ideal harbor for transgenes in hESCs.

## Introduction

Over the past thirty years, the combined use of murine embryonic stem cells (mESCs) and gene targeting, which allows researchers to study gene function *in vivo*, has revolutionized developmental research [1]. Parallel to mESCs, human embryonic stem cells (hESCs) can also proliferate indefinitely and differentiate into multiple lineages both *in vitro* and *in vivo* [2]. More importantly, because they have a human genetic background, they may be powerful tools in the study of human genes and in regenerative medicine. Unfortunately, the gene targeting strategy that is most widely used to manipulate mESCs has worked poorly in hESCs due to their resistance to non-viral transfection and sensitivity to single-cell cloning. So far, only a dozen sites have been successfully targeted in hESCs [3]–[15]. Random transgene integration is the method of genetic modification most commonly used with hESCs; but the uncertainty of the integration site leads to other problems. A transgene may become silenced if it is integrated into a heterochromatin area, or it may disrupt or activate endogenous genes, leading to apoptosis or otherwise changing the cell’s fate. In addition, because it is thought that random integration is mediated by non-homologous end joining (NHEJ), an imprecise DNA-repair mechanism, integration may be incomplete and plasmid debris may end up integrated into the genome [16], [17]. These problems may be solved if the transgene is targeted to an appropriate site where it can be expressed without any serious functional consequences [18].

Human 45S ribosomal DNA (rDNA) is clustered on the short arm of all five acrocentric chromosomes (chromosomes 13, 14, 15, 21, and 22). It consists of approximate 400 copies of the 45S pre-RNA (rRNA) gene per haploid [19]. The rRNA gene is transcriptionally active, producing approximately 80% of the total RNA in rapidly dividing cells. Loss or gain of the short arm of acrocentric chromosomes is common in humans and does not usually have any phenotypic effects and can be inherited stably. Balanced translocations between the short arm of an acrocentric chromosome and the other chromosomes are also observed without apparent phenotypic effect. For this reason, we presumed that transgenes targeted into the rDNA locus would be transcriptionally active without any unexpected effects. The rDNA locus is a candidate harbor favorable for effective and safe transgene cell manipulations. A recent report revealed that the rDNA cluster exhibited strikingly variable lengths between and within human individuals and showed high intrinsic recombinational instability during both meiosis and mitosis [20]. This indicates that a high rate of gene targeting may be achieved. The transgene targeted into the rDNA locus is expected to work in hESCs.

In this report, we targeted human coagulation factor IX, a physiological gene, into the rDNA locus via homologous recombination and found the process to be highly efficient. We here demonstrated that the integrated clones retained the main characteristics of ES cells. After long-term culture, the targeted cells retained a normal karyotype and expressed the transgene stably. Our findings provide a new strategy for manipulating hESCs in both basic and applied research.

## Results

**Figure 1:**
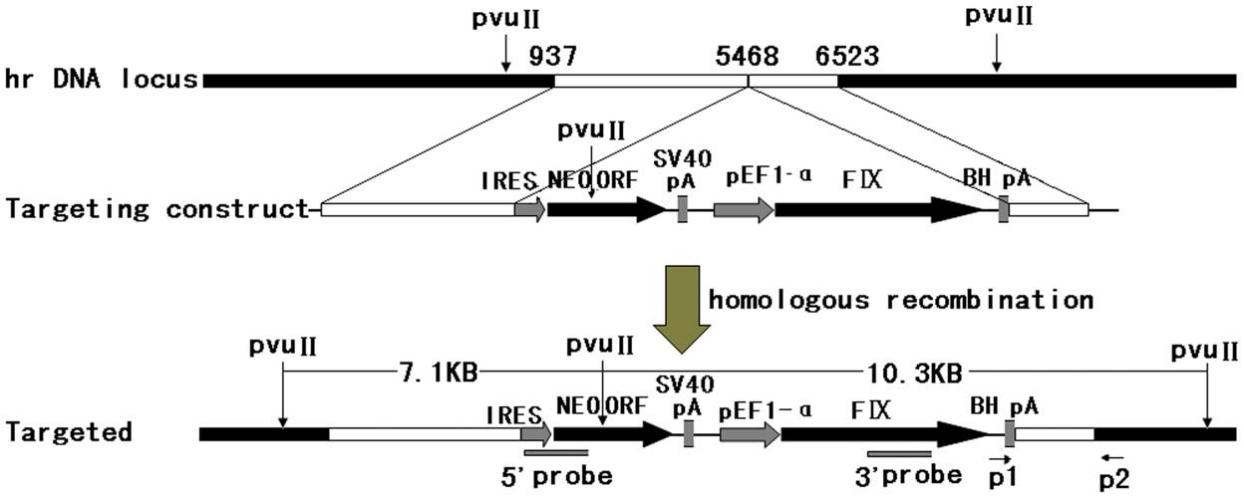
Schematic representation of the rDNA unit, targeting vector, and targeted allele after homologous recombination. White boxes represent the left (GenBank U13369: 937–5467) and right (GenBank U13369: 5468–6523) homologous arms. The neo cassette consisted of an IRES element from the encephalomyocarditis virus, the coding region of the neo gene (NEO), and SV40 polyA signal (SpA). Neo lacks a promoter. Expression is activated by the promoter of the rRNA gene after homologous recombination. Knock-in of *neo* open reading frame (NEO ORF) to the rDNA unit caused an addition of *PvuII* site resulting in fragments of 7.1 kb and 10.3 kb after digestion. The F9 gene is driven by an EF1-αpromoter. Primer 1 (P1) and primer 2 (P2) bind to the bovine growth hormone polyA (BH pA) and the 5.8S RNA coding sequence beyond the homologous sequence respectively, the PCR product will be 1.4 k after homologous recombination.

We constructed an rDNA-targeting plasmid, pHrnF9, which introduced a promoterless neomycin resistance (neo) cassette and an EF1-α driven human coagulation factor IX (*F9*) open reading frame into the 45S pre-RNA gene. The two cassettes were flanked by a 5′ long homologous arm (4.5 kb) and a 3′ short homologous arm (1.1 kb). The first cassette contained an encephalomyocarditis virus internal ribosomal entry site (EMCV-IRES), which enabled resistant gene expression under the control of endogenous RNA polymerase I (Pol I) promoter upstream after homologous recombination (Figure 1).

Prior to targeting hESCs, we tested the new plasmid construct in HT1080 cells via electroporation. We did two gene targeting experiments on HT1080 cells (Table S1). In one of the experiments, three million HT1080 cells were exposed to a single 2000 V, 50 µF pulse with 20 µg linearized pHrnF9 at room temperature using the BioRad Gene Pulser II (0.4 cm gap cuvette, BioRad, Hercules, CA, U.S.). Up to 400 µg/ml of G418 (Sigma, St Louis, MO, U.S.) was added 72 hours after electroporation. Finally, 997 resistant clones were obtained (Table S1). Out of the 39 clones screened by PCR, 12 were identified as homologous recombinants. This indicated a relative targeting efficiency of 31% and an absolute targeting frequency of 0.01%. These values are higher than those of any previous gene targeting experiment performed on HT1080 cells. The targeted clones secreted F9 protein stably (Table S2).

**Figure 2:**
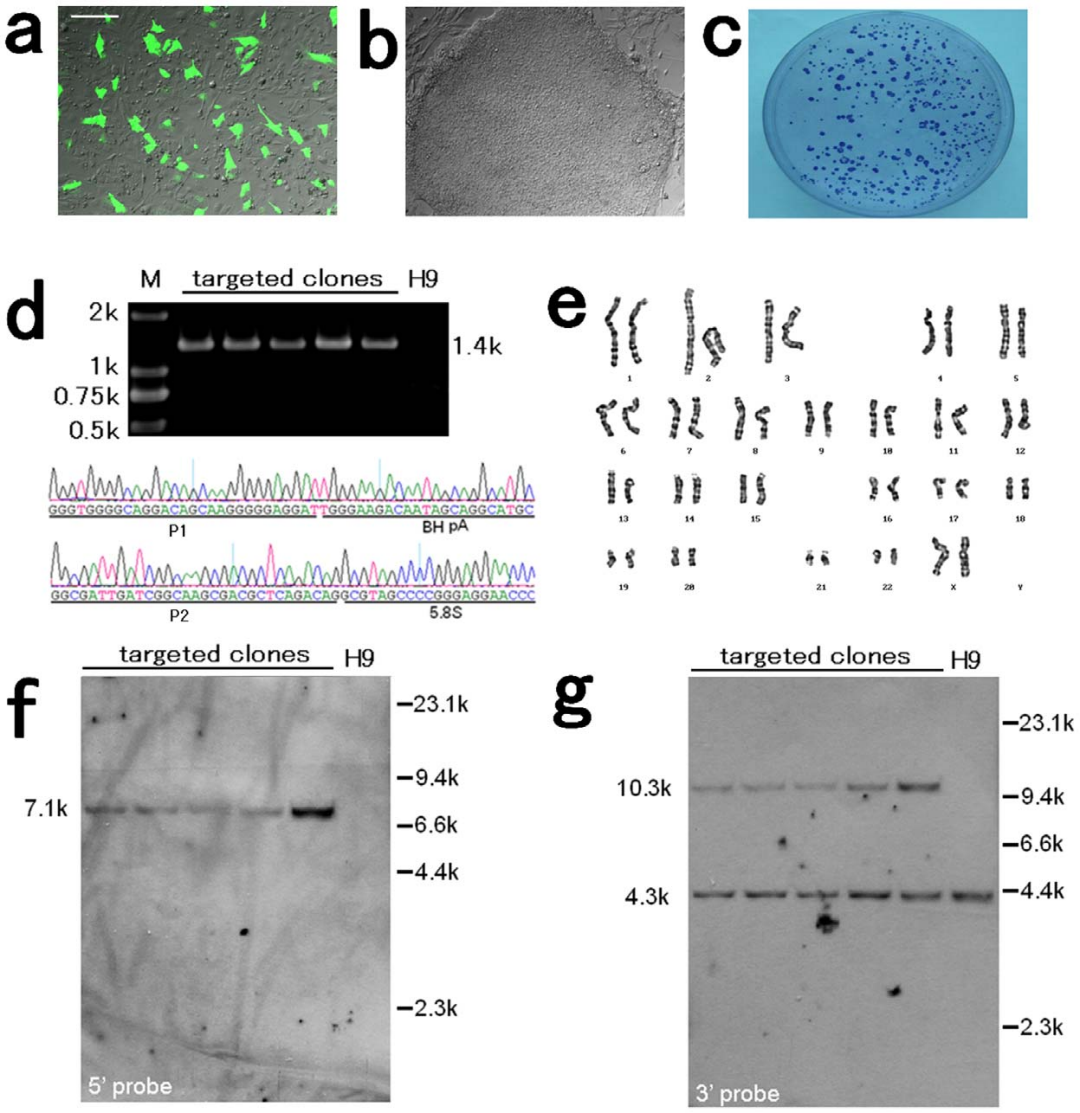
Gene targeting of the rDNA locus in hESCs. (**a**) For hESC transfection, the efficiency was determined by transient nucleofection of H9 single cells by pmaxGFP. (**b**) Phase-contrast image of a resistant clone just before picking up. (**c**) After two weeks of drug selection, a portion of resistant clones were picked up and the remaining clones were fixed and stained with Giemsa stain. Clones with diameters of ≥2 millimeters were considered resistant. (**d**) PCR result using P1 and P2, and DNA sequencing of the PCR product. (**e**) The targeted clones showed a normal karyotype (46, XX). (**f–g**) Southern blot analysis showed a 7.1 kb band for targeted clones using a 5′ probe. When using a 3′ probe corresponding to the second exon of *F9* gene, both a wild type 4.3 kb fragment and an integrated 10.3 kb fragment will be detected. M, marker. Scale bar  = 200 µm (a–b).

For gene targeting of hESCs, we nucleofected trypsinized H9 hESCs, using 10 µM rho-associated kinase (ROCK) inhibitor Y-27632 two hours before and during the 24 hours immediately after nucleofection to improve single-cell survival. This method yielded sufficiently high transfection efficiency (Figure 2a). G418 selection was initiated 48–72 hours after transfection, starting at low drug concentrations and slowly building up to 50 µg/ml. Because H9 cells are highly sensitive to G418, the initial drug concentration of 25 µg/ml was sufficient to kill untransfected cells. Drug-resistant clones were picked and expanded for genotyping two weeks after nucleofection. In a typical experiment, 3.2×10^6^ cells were nucleofected with 5 µg linearized pHrnF9, and 57 drug-resistant clones were obtained. Of the 22 clones analyzed, 14 targeted clones were identified by PCR (Figure 2d) and Southern blot analysis (Figures 2f and 2g). Relative targeting efficiency was 64% (14/22) and the absolute homologous recombination frequency was 1.13×10^−5^. Both of these values are higher than those reported in previous studies other than studies of artificial zinc finger nucleases (ZFNs). Generally, dozens of clones were obtained for each case of single nucleofection followed by two weeks of selection. In a series of experiments, similar results were obtained regularly (Table 1).

**Table 1:** Summary of the 4 experiments of gene targeting in H9 cells.

Exp.	N	C	S	T	R	ATF	RTE
1	1.8	32	23	13	10	9.9	56%
2	1.9	45	21	10	11	11.6	48%
3	3.2	57	22	14	8	11.3	64%
4	2.8	40	22	15	7	9.8	68%
Total	9.7	174	88	52	36	10.6	59%

Abbreviation: Exp., experiments were performed. N, Number of cells nucleofected (×10^6^). C, total number of resistant clones obtained from each experiment. S, number of clones screened. T, number of clones screened as targeted recombinants. R, number of clones screened as random integrants. ATF, absolute targeting frequency (×10^−6^)  =  TC/NS. RTE, relative targeting efficiency = T/S.

**Figure 3:**
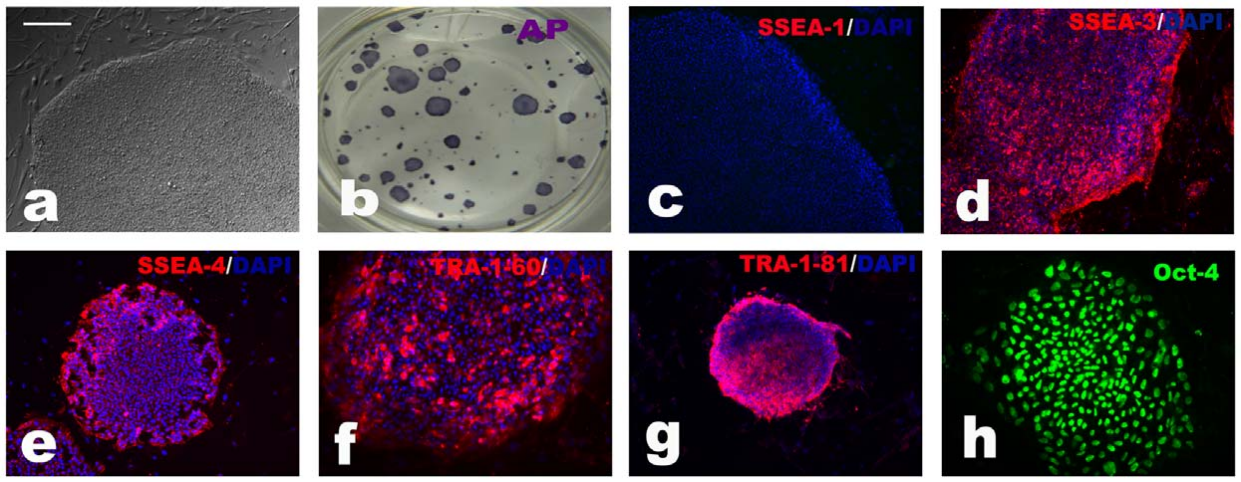
Characterization of targeted clones. (**a**) Phase-contrast image of a targeted clone. (**b**) Alkali phosphatase staining of targeted clones. (**c**–**h)** Immunocytochemical analysis of targeted clones with SSEA-1, SSEA-3, SSEA-4, Tra-1-60, Tra-1-81, and Oct4 antibody. Scale bar  = 200 µm for micrographs.

**Figure 4:**
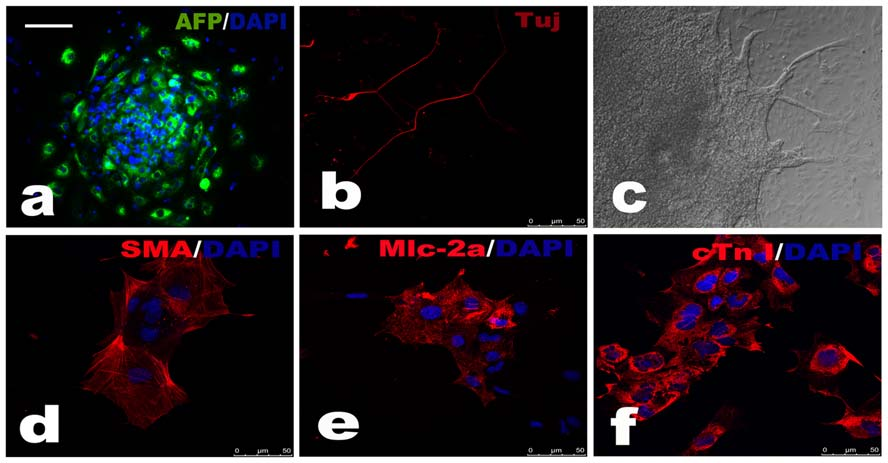
*In vitro* differentiation of targeted clones. Immunostaining images show cells derived from all three germ layers, including AFP (endoderm), Tuj1 (ectodermal), and SMA (mesodermal) positive cells. Upon directed differentiation, cell clumps started beating rhythmically, and the expression of Mlc-2a and cTn I revealed that the differentiation into cardiomyocytes in these cells had been completed. Scale bar  = 200 µm (a, c).

Following successful gene targeting of hESCs, we analyzed G-banded chromosomes on nine targeted clones after genotyping, and all showed normal karyotypes (Figure 2e). To confirm that the targeted clones retained hESC characteristics after gene targeting, we examined hESC marker expression. Immunocytochemistry and alkali phosphatase (AP) staining revealed that the targeted hESCs lines expressed stage-specific embryonic antigen (SSEA)-3 (Figure 3d), SSEA-4 (Figure 3e), tumor-related antigen (TRA)-1-60 (Figure 3f), TRA-1-81 (Figure 3g), OCT 3/4 (Figure 3h), and AP (Figure 3b). SSEA-1 was not expressed in undifferentiated cells (Figure 3c). To determine the differentiation potential of the targeted clones, we cultured the cells in suspension to form embryoid bodies (EBs). After 7 days, the EBs were transferred to gelatin-coated plates and cultured for another 7 days. Immunofluorescent analysis showed that the EBs can give rise to Tuj1-positive ectoderm (Figure 4b), α-fetoprotein (AFP)-positive endoderm (Figure 4a), and mesoderm positive for smooth muscle actin (SMA) (Figure 4d). Upon directed differentiation, beating clumps emerged (Figure 4c).These consisted of cadiocytes expressing myosin light chain-2a (Figure 4e) and cardiac troponin I (Figure 4f).

**Figure 5:**
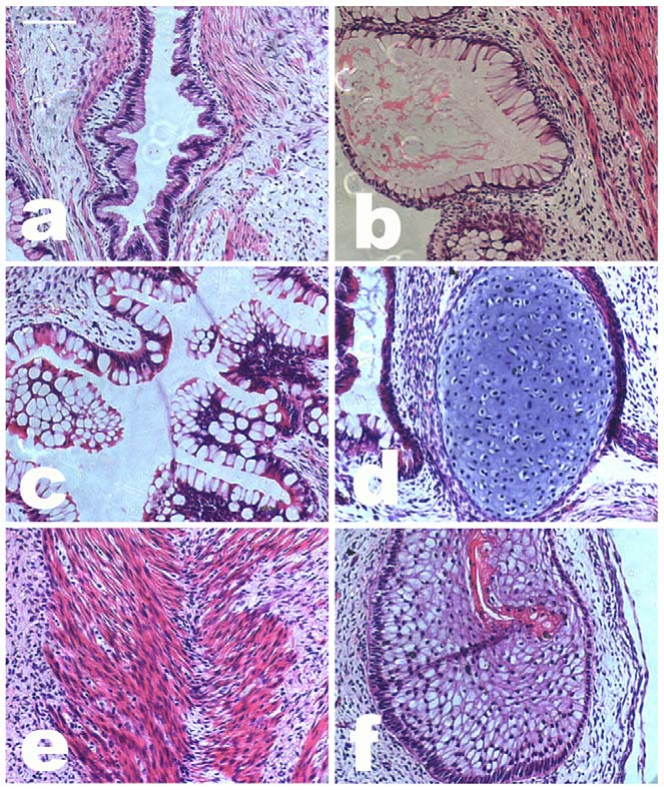
Teratoma formation in immunodeficiency mice by targeted cells. H&E staining of teratomas was performed. Derivatives of all three germ layers were observed in the endoderm: (**a**) gallbladder, (**b**) intestinal-like epithelium, and (**c**) respiratory epithelium; in the mesoderm, (**d**) cartilage and (**e**) muscle; and in the ectoderm, (**f**) squamous epithelium. Bar  = 200 µm.

To evaluate pluripotency *in vivo*, we injected the targeted cells subcutaneously into immuno-compromised mice. Two months later, the targeted cells generated various complex teratomas comprising structures and tissues derived from the three embryonic germ layers, including gallbladder (endoderm) (Figure 5a), colon (endoderm) (Figure 5b), respiratory epithelium (endoderm) (Figure 5c), cartilage (mesoderm) (Figure 5d), striated muscle (mesoderm) (Figure 5e), bone (mesoderm), and squamous epithelium (ectoderm) (Figure 5f). Our data indicate that the rDNA-targeted clones retained their ES characteristics.

**Figure 6:**
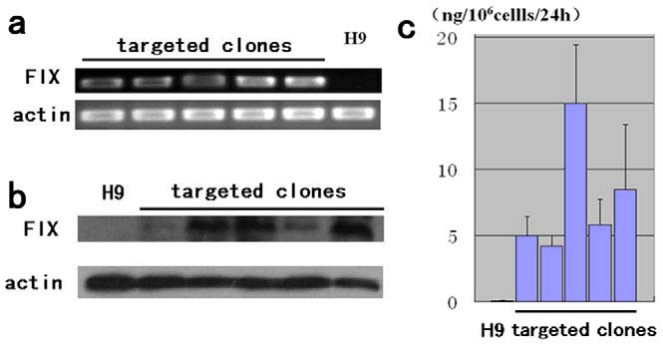
Expression of the transgene in targeted cells. (**a**) RT-PCR analysis of F9expression in targeted clones. The expected product was amplified from all analyzed homologous recombinants after long-term culture (more than 30 passages), while no transcript was detected in wild-type H9 cells. (**b**) Western blot of the clone’s lysate using an antibody anti human F9 protein. (**c**) ELISA analysis of supernatants from recombinant culture. No F9 secretion was detected from wild-type H9 cells. The targeted clones secreted the protein at different levels.

To determine whether targeted transgene could be expressed at the rDNA locus, reverse transcription PCR (RT-PCR) analysis was carried out, revealing that wild-type H9 did not express endogenous *F9* and that the targeted clones did, even after 40 passages of contiguous culture (Figure 6a). Quantitative analysis using enzyme linked immunosorbent assay (ELISA) showed that the targeted clones expressed the transgene at different levels (Figure 6c), indicating the site-specifically integrated transgene could be expressed in the targeted clones. It also showed that the synthesized protein could be secreted into the supernatant. Western blot analysis showed that the F9 protein could be detected in both the cell lysate and concentrated supernatant (Figure 6b and Figure S1). The different levels of transgene expression observed among different targeted clones may have been caused by the different targeted rDNA copies, which had different transcriptional levels.

## Discussion

In recent years, hESCs have been studied eagerly by thousands of laboratories, but results have been disappointing because of their low rate of transgenesis and homologous recombination relative to mESCs. One of main characteristics of hESCs is the maintenance of normal karyotypes after long-term culture. To achieve this, they must maintain some kind of self-repairing mechanism (such as homologous recombination) through hundreds of mitotic divisions. For this reason, we think the intrinsic frequency of homologous recombination may not be as low as reported and the real barrier to hESC gene targeting is their resistance to clonal expansion. This problem can be solved by using of neurotrophin cocktail or ROCK inhibitor [21], [22]. For instance, once treated with Y27632, the survival rate of single hESCs can be improved by more than two orders of magnitude without sacrificing any ES properties [22]. By this means, we achieved homologous recombination rate of more than 10^−5^ in hESCs. In other studies, the difficulty of gene targeting has differed from site to site [11]. The addition of Y27632 can only improve clonal expansion, which directly results in increased numbers of drug-resistant clones; but this does not, in theory, markedly alter the ratio of homologous recombinants to non-homologous recombinants. Normally the targeting efficiency of both mESCs and hESCs is about 1%, but in our experiments, it was more than 50%. The gene-trap strategy that we used can partially explain this, especially considering that this strategy has been reported to be more effective than positive-negative selection [3], [23]. However, the main reason would seem to be the relatively high intrinsic activity of homologous recombination at the rDNA locus. Although there are about 400 copies of rDNA repeats per haploid, it is, to our knowledge, uncertain whether the high frequency of homologous recombination is due to the copy number of target sites. A previous study proved that targeting does not depend on the number of targets in mammalian cells [24]. However, recent study using ZFNs did obtain higher rate of gene targeting in cells with two target sites than in cells with only one [25].

ZFNs have been widely used to achieve efficient homologous recombination of gene targeting vectors with various endogenous loci in cultured and primary mammalian cells [26], even those with very short homologous arms [27]. The customized artificial nucleases can introduce DNA double strand breaks at target sites and then stimulate the cell’s endogenous homologous recombination machinery. In this system, a donor DNA can replace any lost portion of its corresponding chromosomal segment at high efficiency. In 2009, two research groups successfully performed gene targeting in hESCs using ZFNs. Hockemeyer et al. obtained 40 homologous recombinants among 42 resistant clones (relative targeting efficiency 94%) using their most efficient ZFN pair [28].An absolute targeting frequency of 0.14–0.24% was achieved by Zou *et al.* [10]. ZFNs are usually superior when used to generate a mixed population of targeted and untargeted cells without drug selection. However, the use of ZFNs can be compromised by their laborious design process, their toxicity, and other undesired effects. In gene targeting of hESCs with the purpose of generating site-specific edited recombinants, our strategy described here is more effective and safer than the ZFNs method.

Human *ROSA26* and *ENVY* loci are transcriptionally active and considered to be candidate sites for targeted transgene integration [5], [29]. But it is uncertain whether the cells will exhibit functional consequences when one of the two copies is disrupted. Another hESC line for gene targeting has been reported. In this line, a LoxP-docking site was introduced in to the *HPRT* locus [12]. This cell line can be used for targeting exogenous sequences using the Cre-Lox system. Because the first round of targeting is favorable to an active X chromosome, disruption of the *HPRT* locus may lead to a Lesch-Nyhan phenotype. In contrast, the human rRNA cluster consists of hundreds copies of tandemly repeated rDNA units. Variations in rDNA copy number are common among healthy individuals and balanced chromosomal translocation involving the rRNA cluster occurs without any apparent phenotypic effect. These properties indicate that the rDNA locus may hold a high intrinsic homologous recombination activity and facilitate effective transgene expression. In the present study, the rDNA locus was targeted with high efficiency, no undesired effect was detected, and the transgene was expressed stably in the integrated clones.

It has been reported that RNA polymerase II (Pol II)-transcribed genes can be silenced at the rDNA locus in yeast cells, especially under an active Pol I promoter [30], [31]. In the current study, we screened the resistant clones using a promoter-trap strategy. This means that the neo gene could only be expressed under a transcribed Pol I promoter. As a result, the clones cannot be selected unless the transgene is targeted into an active rDNA repeat in a dynamic chromatin structure. However, we found that all the five selected clones expressed the Pol II gene. This differs from the results of a previous study conducted in yeast. The question raised by our results is whether the mechanism of the Pol II gene silencing at the rDNA locus is similar in all eukaryotes, from yeast to human cells. Restructuring of the rDNA clusters has been observed among somatic cells [20]. This raises concerns about the potential instability of any inserted transgene in the rDNA locus and the risk that the transgene might be translocated among the rDNA clusters or lost during mitotic expansion. However, the odds of this happening to one specific targeted copy out of the hundreds of rDNA copies in the human genome are very low. They are rendered even lower by the fact that the restructuring of the rDNA clusters during mitosis is rare.

In summary, using the strategy described here, we integrated human coagulation factor IX into the rDNA locus of hESCs via homologous recombination. Compared to other sites reported, this locus proved to be subject to efficient targeting. The targeting of hESCs at the rDNA locus did not change the main ES characteristics of the cells and the transgene was expressed stably in targeted hESCs. This is the first gene targeting of a human physiological gene at a defined locus on hESCs. Our findings indicate that the rDNA locus may serve as an ideal harbor for transgenes in hESCs.

## Materials and Methods

### Cell Culture

Human fibrosarcoma cells (HT1080) were purchased from ATCC and cultured in high-glucose Dulbecco’s modified Eagle’s medium (4.5 g/l) supplemented with 10% fetal bovine serum,2 mM L-glutamine, 0.1 mM non-essential amino acids, 50 unit/ml penicillin, and 50 mg/ml streptomycin at 37°C in a humidified 5% CO_2_atmosphere. H9 cells (WiCell Research Institute, Madison, WI, U.S.) were maintained on mitomycin-C treated mouse embryonic fibroblasts in hESC medium containing DMEM/F12 supplemented with 20% knockout serum replacement, 2 mM L-glutamine, 1% non-essential amino acids, 0.1 mM β-mercaptoethanol, 100 unit/ml penicillin and 100 mg/ml streptomycin and 10 ng/ml basic fibroblast growth factor.All components were purchased from Invitrogen. Cells were passaged every five days mechanically or through dispase (1 mg/ml, Invitrogen, Carlsbad, CA, U.S.) digestion.

### Construction of Plasmid and Gene Targeting

Human 45S pre-RNA genes are arranged as tandem repeat clusters on the satellite stalks (p12) of the five pairs of acrocentric chromosomes (Chromosome 13, 14, 15, 21 and 22). All the primers were designed according to the human ribosomal DNA complete repeating unit (GenBank accession number U13369). Homologous sequences were amplified from normal human genomic DNA with the following primers:5′-TTC AAT TGC GGT GTG GGG TTC GAG GCG GTT TGA GTG AGA CG-3′/5′-CCA AGT AGG AGA GGA GCG AGC GAC CAA AGG AAC CAT AAC TG-3′;5′-GGG GCT CGC CGC GCT CTA CCT TAC CTA CCT G-3′/5′-GCC GAT CCG AGG GCC TCA CTA AAC CAT CCA A-3′; 5′-CTG AAA CTT AAA GGA ATT GAC GGA AGG GCA CCA CCA GGA GTG-3′/5′-GGG TTG CCT CAG GCC GGC CAG ACG AGA CA-3′.The Factor IX open reading frame and the 3′untranslated region were generated using PNS-FIX as templates with the following primers: 5′-GGC GTC TCA CAT GCA GCG CGT GAA CAT GAT -3′/5′- CCG CTA GCT ACC CCC TAG AGC CCC AGC- 3′.These introduced a Bsmb I site into the product. Neo open reading frame, 3′untranslated region and SV40 Poly A sequence were generated using pCDNA3.1 as a template with primers 5′-GGC GTC TCA CAT GAT TGA ACA AGA-3′/5′-CCA TGG CTA GCT CTA GAC GGT CGA CCC GTG CGG AAT GCT TCC GGC TCG TAT GTT GTG T-3′ which introduced human rRNA gene terminator and recognition sites for NcoI, XbaI, and NheI.EIF4G-IRES was amplified from human normal genomic DNA using primers 5′-GAA TTC TCT AGA TGG GGG TCC TGG GC-3′/5′-TCC TCC TTG GTT GGG ATC TCG-3′ as reported [32]. All the PCR above were performed using Pyrobest ™ DNA Polymerase (Takara, Dalian, China).

For gene targeting, one passage prior to transfection, hESCs were detached by 1 mg/ml dispase and replated on dishes coated with Matrigel™ (BD Biosciences, San Jose, CA, U.S.) for three days in MEF conditioned hESC medium. Two hours before transfection, 10 µM Y27632 was added to the medium. For targeting, the hESCs were incubated at 37°C with trypLE™ Select (Invitrogen, Carlsbad, CA, U.S.) for 3 minutes. Then the cells were immediately collected and counted. The centrifuged cells were resuspended with 100 µL Human Stem Cell Nucleofector Kit 2 (Lonza, Basel, Switzerland) and 5 µg linearized pHrnF9 and nucleofected using Nucleofector II (Lonza, Basel, Switzerland) using program A023. After recovery in 500 µL RPMI-1640 (Hyclone, Beijing, China) for 5 minutes at room temperature, the transfected cells were plated on PMEF-NL (Millipore, Bedford, MA, U.S.) in hESC medium containing 10 µM Y27632 (Sigma, St Louis, MO, U.S.). G418 selection was initiated 48–72 hours after transfection, depending on the cell density. The final concentration of G418 was 50 µg/ml. About two weeks after transfection, a portion of resistant clones was picked and the remaining clones were fixed and stained with Giemsa stain. Clones with diameters of ≥2 mm were considered resistant.

### PCR and RT-PCR

Genomic DNA was isolated from the cells using phenol/chloroform extraction. The primers used are as follows: P1 5′- GGG TGG GGC AGG ACA GCA AGG GGG AGG AT -3′; P2 5′- GGC GAT TGA TCG GCA AGC GAC GCT CAG ACA G -3′.

Total RNA was extracted using Trizol reagent (Sigma, St Louis, MO, U.S.) and reverse transcribed using Promega’s transcription system according to the manufacturers’ instructions. RT-PCR for F9 was performed using primers 5′- ATG CAG CGC GTG AAC ATG A-3′ and 5′- TAC CTC TTT GGC CGA TTC AGA -3′.

### Southern Blotting

After digested with pvuII restriction enzyme (New England Biolabs, Ipswich, MA, U.S.) overnight, 5 µg genomic DNA were electrophoresed on a 0.8% agarose gel over night then transferred to positively charged nylon membranes (Roche Diagnostics, Indianapolis, IN, U.S.). λ DNA Hind III (Takara, Dalian, China) was used as molecular weight marker. The blots were hybridized with DIG-dUTP labeled probes overnight at 42°C. After incubation with AP-conjugated DIG-Antibody (Roche Diagnostics, Indianapolis, IN, U.S.) and appropriate washing, the signals were detected using CDP-Star (Roche Diagnostics, Indianapolis, IN, U.S.) as a substrate for chemiluminescence. Probes were generated by PCR DIG Probe Synthesis Kit (Roche Diagnostics, Indianapolis, IN, U.S.) using the primers: 5′ probe 5′- CCC GGA AAC CTG GCC CTG TCT T-3′ and 5′-TGG GGT ACC TTC TGG GCA TCC TTC-3′; 3′ probe 5′-GCT CCA TGC CCT AAA GAG AA -3′and 5′-TCC ATC AAC ATA CTG CTT CCA-3′.

### Karyotyping

Three-day old cell clumps were treated with 0.08 µg/ml colcemid (Sigma, St Louis, MO, U.S.) for 2.5 hours. Then the cells were tripsinized, centrifuged, and incubated in 0.075 M KCl for 30 minutes at 37°C. After fixing with Carnoy fixative, metaphase chromosome spreads were prepared using air drying.

### Alkaline Phosphatase Staining and Immunofluorescence

Three day old cell clumps were fixed with 4% paraformaldehyde. After washing in TBST, alkaline phosphatase was stained with 0.375 mg/ml nitrobluetetrazolium chloride and 0.188 mg/ml 5-bromo-4-chloro-3-indolyl-phosphate (Roche, Indianapolis, IN, U.S.), pH 9.5, for 15 minutes in darkness for detection of alkaline phosphatase. For immunofluorescent staining, cells on gelatin-coated coverslips were fixed in 4% paraformaldehyde and permeabilized with 0.1% Triton-X 100. After blocking with 10% normal donkey serum (Jackson ImmunoResearch, West Grove, PA, U.S.), the cells were incubated with the first antibody against Oct4 (1∶200, Millipore, Bedford, MA, U.S.), SSEA-1 (1∶100, Millipore,Bedford, MA, U.S.), SSEA-3 (1∶100, Millipore, Bedford, MA, U.S.), SSEA-4 (1;200, Millipore,Bedford, MA), Tra-1-60 (1∶100, Millipore, Bedford, MA, U.S.), Tra-1-81 (1∶100, Millipore, Bedford, MA, U.S.), SMA (1;200, Millipore, Bedford, MA, U.S.), AFP (1∶100, Millipore, Bedford, MA, U.S.), Tuj1 (1∶300, Sigma, St Louis, MO, U.S.), c Tn I (1∶100, Millipore, Bedford, MA, U.S.), and Mlc-2a (1∶200, Synapric Systems, Goettingen, Germany) at room temperature for 1 hour. The samples were incubated with appropriate secondary antibodies after triple washing. DNA was visualized using DAPI (Sigma, St Louis, MO, U.S.).

### *In vitro* Differentiation

Three day old cell clumps were incubated with 1 mg/ml dispase for 10 minutes at 37°C then washed with DMEM/F12. After culture on ultra-low attachment plates for 7 days in hESC medium without bFGF, EBs were transferred to gelatin-coated coverslips and cultured for another 7days. Differentiated cells were analyzed by immunofluorescence. Directed differentiation was performed according to the protocol of the National Stem Cell Bank of America (https://www.wicell.org/index.php?option=com_docman&task=doc_download&gid=1064).

### Teratomaformation and Analysis

Targeted cells from one 10 cm dish were dissociated with 0.05% trypsin/EDTA (Invitrogen) and collected in DMEM/F12. After being washed with DMEM/F12, cells were resuspended in 140 µL DMEM/F12 and 70 µL Matrigel™ (BD Bioscience, San Jose, CA, U.S.). The suspension was injected subcutaneously into the hind legs of immunocompromised mice. Eight to twelve weeks later, the formed teratomas were removed and fixed in 4% paraformaldehyde overnight. The fixed tissues were sectioned and stained with hematoxylin and eosin. All procedures regarding the care and use of animals are in accordance with institutional guidelines. This study was approved by the Ethics Committee of State Key Laboratory of Medical Genetics of China, No. 2008-ANIMAL-004.

### Western Blot

Protein samples were electrophoresed and electrotransferred onto PVDF membranes (Millipore, Bedford, MA, U.S.). Blots were incubated with a primary antibody anti-human Factor IX (Affinity Biologicals, Canada) overnight at 4°C. The blots were than incubated with horseradish peroxidase-conjugated secondary antibodies(Sigma, St Louis, MO, U.S.) for 1 hour at room temperature and detected with an ECL system (Amersham Biosciences, Piscataway, NJ, U.S.). Prestained molecular weight standards (Fermentas, Glen Burnie, MA, U.S.) were used to estimate the apparent molecular weight.

### ELISA

After culturing in hESC medium for three days, 24-hour-old supernatants were collected from six-well plates. Total cells (together with MEFs) and MEFs from parallel wells (subtracting the number of MEFs from the total number of cells) were trypsinized and counted. All supernatants were collected in triplicate. ELISA was performed using Paired Antibodies for ELISA-Factor IX (Cedarlane Laboratories, Ltd., Burlington, Canada) according to the manufacturer’s instructions. Reference curves were constructed using serial dilutions of normal pooled plasma (Pacific Hemostasis, Cape Town, South Africa), with correlation coefficient (R^2^) of at least 0.990 using a 5-parameter logistic curve fit algorithm.

## Supporting Information

Figure S1 Western blot analysis of the concentrated supernatant from targeted hES clones.
(TIF)

Table S1 Summary of the 2 experiments of gene targeting in HT1080 cells.
(DOC)

Table S2 FIX levels in cultured supernatant from targeted HT1080 clones.
(DOC)
