# Supplementary figures and images for: Targeting of the Human Coagulation Factor IX Gene at rDNA Locus of Human Embryonic Stem Cells
Source: PLoS One. 2012 May 16;7(5):e37071. doi: 10.1371/journal.pone.0037071 (PMC3353886; doi:10.1371/journal.pone.0037071)

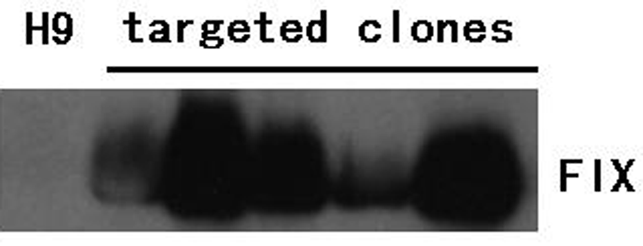

Supplement: Figure S1 — Western blot analysis of the concentrated supernatant from targeted hES clones. (TIF) [file pone.0037071.s001.tif]
