# Supplementary material for: Targeting of the Human Coagulation Factor IX Gene at rDNA Locus of Human Embryonic Stem Cells
Source: PLoS One. 2012 May 16;7(5):e37071. doi: 10.1371/journal.pone.0037071 (PMC3353886; doi:10.1371/journal.pone.0037071)
Supplement: Table S1 — Summary of the 2 experiments of gene targeting in HT1080 cells. (DOC) [file pone.0037071.s002.doc]

Table S1. Summary of the 2 experiments of gene targeting in HT1080 cells

| Exp. | N | C | S | T | RTE | ATF |
| --- | --- | --- | --- | --- | --- | --- |
| 1 | 2.86×10^6^ | 1032 | 40 | 17 | 43% | 1.5×10^-4^ |
| 2 | 3×10^6^ | 997 | 39 | 12 | 31% | 1.0×10^-4^ |

Abbreviation: Exp., experiments were performed. N, Number of cells nucleofected. C, total number of resistant clones obtained from each experiment. S, number of clones screened. T, number of clones screened as targeted recombinants. RTE, relative targeting efficiency =T/S. ATF, absolute targeting frequency = TC/NS.
