# Supplementary material for: Targeting of the Human Coagulation Factor IX Gene at rDNA Locus of Human Embryonic Stem Cells
Source: PLoS One. 2012 May 16;7(5):e37071. doi: 10.1371/journal.pone.0037071 (PMC3353886; doi:10.1371/journal.pone.0037071)
Supplement: Table S2 — FIX levels in cultured supernatant from targeted HT1080 clones. (DOC) [file pone.0037071.s003.doc]

Table S2. FIX levels in cultured supernatant from targeted HT1080 clones.

| Clone Number | FIX Level（ng/10^6^cells/24hr） | Clone Number | FIX Level（ng/10^6^cells/24hr） |
| --- | --- | --- | --- |
| F9 1-9-1 | 42.3 | F9 2-20-1 | 78.3 |
| F9 1-11-1 | 79.8 | F9 2-3-2 | 8.3 |
| F9 1-13-1 | 17.8 | F9 2-4-2 | 61.5 |
| F9 1-15-1 | 71.8 | F9 2-16-2 | 33.3 |
| F9 1-18-1 | 30.5 | F9 1-17-2 | 26 |
| F9 1-19-1 | 39.3 | F9 1-19-2 | 14.5 |
| F9 1-20-1 | 0 | F9 2-4-1 | 135.8 |
| F9 1-1-2 | 38.5 | F9 2-7-1 | 60.3 |
| F9 1-4-2 | 12 | F9 2-9-1 | 61 |
| F9 1-5-2 | 35.3 | F9 2-10-1 | 79 |
| F9 1-10-2 | 59.3 | F9 2-11-1 | 102.8 |
| F9 1-11-2 | 13 | F9 2-14-1 | 0 |
| F9 1-14-2 | 584.5 | F9 2-16-1 | 125.5 |
| F9 1-15-2 | 29.5 | F9 2-18-1 | 463.3 |
| F9 1-16-2 | 37.3 | average | 80.7 |
